# Cortical Projection From the Premotor or Primary Motor Cortex to the Subthalamic Nucleus in Intact and Parkinsonian Adult Macaque Monkeys: A Pilot Tracing Study

**DOI:** 10.3389/fncir.2020.528993

**Published:** 2020-10-26

**Authors:** Simon Borgognon, Jérôme Cottet, Simon Badoud, Jocelyne Bloch, Jean-François Brunet, Eric M. Rouiller

**Affiliations:** ^1^Department of Neurosciences and Movement Sciences, Faculty of Science and Medicine, Section of Medicine, Fribourg Cognition Center, Platform of Translational Neurosciences (PTN), Swiss Primate Competence Center for Research (SPCCR), University of Fribourg, Fribourg, Switzerland; ^2^Center for Neuroprosthetics and Brain Mind Institute, School of Life Sciences, Swiss Federal Institute of Technology (EPFL), Lausanne, Switzerland; ^3^Department of Neurosurgery, Lausanne University Hospital (CHUV), Lausanne, Switzerland; ^4^Cell Production Center (CPC), Lausanne University Hospital (CHUV), Lausanne, Switzerland

**Keywords:** non-human primate, anterograde tracing, motor cortex, basal ganglia, Parkinson

## Abstract

Besides the main cortical inputs to the basal ganglia, *via* the corticostriatal projection, there is another input *via* the corticosubthalamic projection (CSTP), terminating in the subthalamic nucleus (STN). The present study investigated and compared the CSTPs originating from the premotor cortex (PM) or the primary motor cortex (M1) in two groups of adult macaque monkeys. The first group includes six intact monkeys, whereas the second group was made up of four monkeys subjected to 1-methyl-4-phenyl-1,2,3,6-tetrahydropyridine (MPTP) intoxication producing Parkinson’s disease (PD)-like symptoms and subsequently treated with an autologous neural cell ecosystem (ANCE) therapy. The CSTPs were labeled with the anterograde tracer biotinylated dextran amine (BDA), injected either in PM or in M1. BDA-labeled axonal terminal boutons in STN were charted, counted, and then normalized based on the number of labeled corticospinal axons in each monkey. In intact monkeys, the CSTP from PM was denser than that originating from M1. In two PD monkeys, the CSTP originating from PM or M1 were substantially increased, as compared to intact monkeys. In one other PD monkey, there was no obvious change, whereas the last PD monkey showed a decrease of the CSTP originating from M1. Interestingly, the linear relationship between CSTP density and PD symptoms yielded a possible dependence of the CSTP re-organization with the severity of the MPTP lesion. The higher the PD symptoms, the larger the CSTP densities, irrespective of the origin (from both M1 or PM). Plasticity of the CSTP in PD monkeys may be related to PD itself and/or to the ANCE treatment.

## Introduction

In parallel to the multiple descending motor pathways originating from cortical areas, such as the corticospinal (CST), the corticorubral, corticotectal, and the corticobulbar (corticoreticular) projections (Lemon, 2008), motor control is also based on cortico-basal ganglia circuit loops. The majority of motor cortical inputs to the basal ganglia are directed to the striatum (e.g., Künzle, 1975; McGuire et al., 1991; Inase et al., 1996, 1999; Takada et al., 1998a,b; Tokuno et al., 1999; Nambu et al., 2002a; Tachibana et al., 2004; Parent and Parent, 2006; Raju et al., 2008; Gerbella et al., 2016; Innocenti et al., 2017). However, other cortical projections to the basal ganglia reach the subthalamic nucleus (STN), *via* corticosubthalamic projections (CSTPs) originating from the primary motor cortex (M1), the supplementary motor areas (SMA-proper and pre-SMA), the premotor cortex (PM), and the cingulate motor areas, representing a hyper direct pathway parallel to the classic direct and indirect pathways (Nambu et al., 1996, 1997, 2000, 2002b; Inase et al., 1999; Takada et al., 2001; Miyachi et al., 2006; Degos et al., 2008; Inoue et al., 2012; Iwamuro et al., 2017; Coudé et al., 2018; Temiz et al., 2020).

Based on the 1-methyl-4-phenyl-1,2,3,6-tetrahydropyridine (MPTP) intoxication model, producing Parkinson’s disease (PD)-like symptomatic macaque monkeys, it was shown that a cellular therapy referred to as autologous neural cell ecosystem (ANCE) improves recovery of motor control (Bloch et al., 2014; Borgognon et al., 2017, 2019). Subsequently, in four of such MPTP intoxicated monkeys subjected to ANCE therapy, tracing studies showed that the corticobulbar (corticoreticular) projections were significantly reduced as compared to intact monkeys (Fregosi et al., 2018), whereas the corticotectal projection was much less affected (Fregosi et al., 2019). As far as the corticosubthalamic projection (CSTP) is concerned, a loss of the hyper direct pathway was reported in PD mice (Chu et al., 2017). Similarly, the cortical innervation of the STN was reduced in PD monkeys (Mathai et al., 2015). In the latter two studies, the evidence for a reduction of cortical inputs to STN was derived from immunohistochemical detection of vesicular glutamate transporter type-1 terminals, making it impossible to discriminate the distinct origins of the CSTP inputs, for example, M1 vs. PM.

Our goal then was to investigate in 10 adult macaque monkeys how CSTPs originating from PM and M1 are affected by PD-like symptoms, by comparing a group of six intact monkeys with another group of four PD monkeys treated with ANCE. Each group of monkeys was split into two, half of the animals injected with the anterograde tracer biotinylated dextran amine (BDA) in PM and the other half receiving the same injection in M1. It was hypothesized that PD and the subsequent recovery following ANCE treatment, would result in a modification of the density of the CSTP when compared to intact monkeys. Subsequently, the group of intact monkeys was used to investigate whether the density of the CSTP differs based on its origin, PM vs. M1.

## Materials and Methods

All surgical experimental procedures, experiments, and animal care were conducted in respect to the ethical guidelines (ISBN 0-309-05377-3, 1996) and authorized by the local (Canton of Fribourg) and federal (Switzerland) veterinary authorities (authorizations No. 44_92_3; 150_00; 156_02; 156_08E; 2012_01-FR; 2012_01E_FR). The conditions of housing in the animal facility were described earlier in detail (Borgognon et al., 2017, 2019; see also: http://www.unifr.ch/spccr/about/housing).

**Figure 1:**
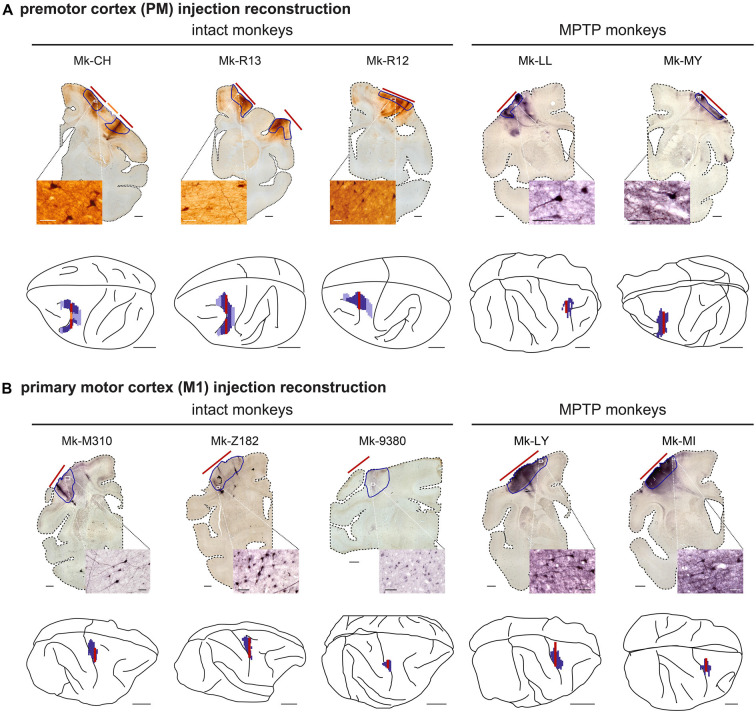
Reconstruction of the biotinylated dextran amine (BDA) injection sites. **(A)** Reconstruction of the animals injected with BDA in the premotor cortex and **(B)** for the animals injected in the primary motor cortex (M1). The microphotographs show examples of one histological frontal section, from which the extent of BDA uptake (red lines) are transposed to the 3D brain (red line). Next to each frontal section, the insets show a photomicrograph at high magnification illustrating BDA-labeled cortical neurons in the vicinity of the core of the injection site. Scale bar = 10 mm for the 3D brains, 2 mm for the frontal sections, and 50 μm for the magnifications microphotographs. Modified from Fregosi et al. (2017); Fregosi et al. (2018).

The materials and methods are similar to those reported in recent publications related to the corticobulbar (corticoreticular) and corticotectal projections (Fregosi and Rouiller, 2017; Fregosi et al., 2017, 2018, 2019). Briefly, surgeries were performed under sterile conditions in anesthetized animals. The protocol consisted of a unilateral cortical (PM or M1) injection of the anterograde tracer BDA (MW = 10,000; Molecular Probe, Eugene, OR, USA) using a 10 μl Hamilton micro-syringe. BDA concentration was 5% (in distilled water) for Mk-M93-80 and 10% for the other nine monkeys (see Table 1). The extent of the BDA injection was assessed on consecutive histological frontal sections, each reconstructed manually using Neurolucida software (MBF Bioscience-MicroBrightField Inc., version 11). Three-dimension (3D) reconstruction was performed by stacking on a dorsolateral view the medio-lateral extent of BDA intake from the midline. The injection sites in PM involved both PMd and PMv in most animals, except in Mk-R12 and Mk-LL in which BDA was delivered mostly in PMd. The BDA injection in M1 was limited to the hand area in Mk-93-80, as defined by intracortical microstimulation. For the other M1 injections, based on anatomical landmarks (cortical sulci), BDA involved the hand area, as well as more proximal territories of the forelimb (Figure 1). Although, some modest spread (so-called “halo”) in the PM injected animals could be observed, BDA in general did not exhibit extensive spread (Fregosi et al., 2017, 2018; see Mk-CH; Mk-R13 and Mk-R12 in Figure 1). Using an exhaustive plotting method (perfectly suitable to the small size of the STN) with Neurolucida software, the axonal terminal boutons in the STN labeled with the anterograde tracer BDA were registered in each monkey, following unilateral BDA injection either in M1 or in PM. The BDA injections took place usually about 30 days before euthanasia. The same 10 monkeys involved in the present analysis (Table 1) were already used to establish the properties of the corticoreticular projection from M1 and PM in intact monkeys (Fregosi et al., 2017) and in PD monkeys (Fregosi et al., 2018), as well as the corresponding corticotectal projections (Fregosi and Rouiller, 2017; Fregosi et al., 2019).

**Table 1:** All monkeys were adult *Macaca fascicularis*.

	BDA injection in PM					BDA injection in Ml				
	Intact monkeys			MPTP monkeys		Intact monkeys			MPTP monkeys	
Monkey’s ID	Mk-R12	Mk-R13	Mk-CH	Mk-MY	Mk-LL	Mk-M310	Mk-Z182	Mk-93-80	Mk-MI	Mk-LY
Sex	female	female	male	female	female	male	male	male	female	female
Age at sacrifice (years)	6	4.5	10	9.5	7.5	8.5	7.5	4	9.5	7.5
Weight (kg)	4	4	6	4.3	3.6	10	10	4	3.3	3.3
BDA volume injected (μl)	7.2	8.8	8	11.5	9.7	22.5	25.5	10.5	9	9
Nb. of BDA injections sites	9	11	10	9	8	15	17	7	6	6
Loss of TH+ neurons in SNpc	-	-	-	−72%	−67%	-	-	-	−74%	−39%
Nb. of boutons (raw)	1,395	1,722	788	1,466	434	109	127	684	541	25
Inter-section distance (μm)	250	250	250	500	500	400	400	350	500	500
Sampling interval	1/2	1/2	1/2	all	all	all	all	1/2	all	all
Correction factor	2	2	2	2	2	1.6	1.6	2.8	2	2
Corrected nb. of boutons	2,790	3,444	1,576	2,932	868	174	203	1,915	1,082	50
Nb. of CST labeled axons	1,473	1,802	1,201	611	593	703	950	3,195	1,117	1,671
Norm and Corr. of boutons	**1,894**	**1,911**	**1,312**	**4,799**	**1,464**	**248**	**214**	**599**	**969**	**30**
MD perf. post-MPTP	-	-	-	72%	100%^&^	-	-	-	13%	100%
MD perf. post-ANCE	-	-	-	95%	100%^&^	-	-	-	52%	100%

*All 1-methyl-4-phenyl-1,2,3,6-tetrahydropyridine (MPTP) monkeys were treated with autologous neural cell ecosystem (ANCE). The inter-section interval of 250 microns was taken as a reference to compute the correction factor (see “Materials and Methods” section). The number of biotinylated dextran amine (BDA)-labeled axonal boutons in subthalamic nucleus (STN) was corrected for other inter-sections intervals, as well as the sampling interval (all sections analysed or 1 out of 2 sections analyzed; see “Materials and Methods” section). Finally, the corrected number of axonal boutons in STN was normalized, based on the number of corticospinal axons labeled with BDA in the same monkey (see “Materials and Methods” section). The loss of TH+ neurons in the SNpc was expressed in % with respect to intact monkeys (see Borgognon et al., 2017). MD, Manual Dexterity; performance measured based on the Modified Brinkman Board, with reference to the performance pre-lesion (100%). See Borgognon et al. (2019). ^*&*^For Mk-LL, the MD performance was hectic, preventing the calculation of reliable scores, although the deficit post MPTP lesion was rather modest or inexistent. Therefore, her MD performance was considered to be at 100% (see “Materials and Methods” section). Further individual data for these monkeys can be found in Fregosi and Rouiller (2017), Fregosi et al. (2017, 2018, 2019), Badoud et al. (2017) and Borgognon et al. (2017, 2019)*.

The six intact monkeys were restricted to tracing investigations, whereas the four PD monkeys were also included in behavioral, imaging, and histochemical assessments (Badoud et al., 2017; Borgognon et al., 2017, 2019). The low-dose MPTP intoxication (see Mounayar et al., 2007) in the four PD monkeys as well as the ANCE transplantation have been previously described (Borgognon et al., 2017, 2019). Briefly, the MPTP protocol consisted of a series of daily intramuscular injections (Sigma–Aldrich Company; 0.5 mg/kg, dissolved in saline solution) until the monkeys reached an adequate level of motor impairment as assessed by the Schneider MPTP scale (Schneider et al., 1995). During the MPTP intoxication protocol, a dorsolateral prefrontal cortex biopsy was performed to obtain the necessary cellular material (Badoud et al., 2017). Then, the cortical samples were immediately processed to be put into the culture to obtain the ANCE. After the post-MPTP lesion plateau (about 6 weeks after the last MPTP injection), the ANCE were implanted in six striatal regions: one in the caudate nucleus and two in the putamen for both hemispheres. All four animals were trained to perform two fine manual dexterity tasks: (1) the modified-Brinkman board task, where the animal retrieved a food pellet using a precision grip (opposition thumb and index finger); and (2) the reach and grasp drawer task, where the monkey pulled-open a drawer to retrieve a reward using a precision grip. During the entire experimental protocol, all four animals were tested pre-post-MPTP lesion and post-ANCE transplantation.

**Figure 2:**
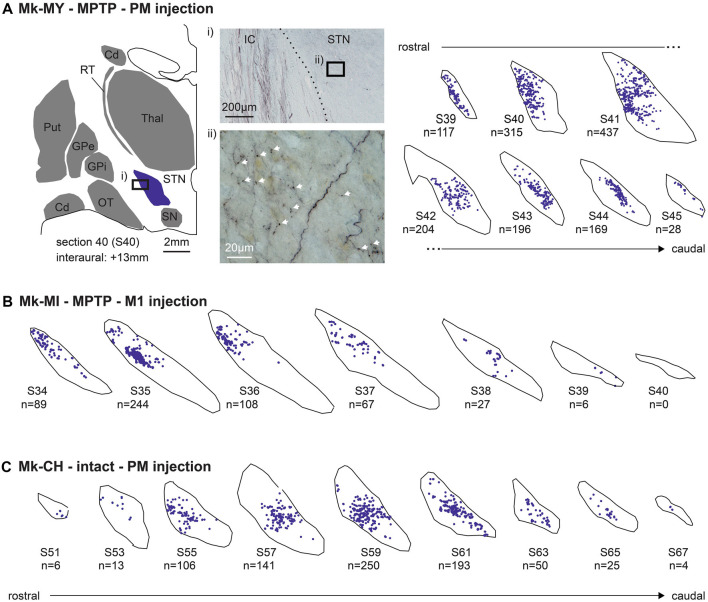
Typical distribution of BDA-labeled corticosubthalamic axonal boutons in the ipsilateral subthalamic nucleus (STN) in three representative monkeys, two subjected to 1-methyl-4-phenyl-1,2,3,6-tetrahydropyridine (MPTP) intoxication [Mk-MY and Mk-MI, **(A)** and **(B)**] and one intact monkey [Mk-CH, **(C)**]. BDA was injected in PM in Mk-MY and Mk-CH, whereas it was injected in M1 in Mk-MI. Axonal boutons are depicted with blue dots on the consecutive sections of STN, from rostral to caudal. **(A)** For Mk-MY, a lower magnification representation of one section (number 40) is shown in order to show the location of STN with respect to other brain structures. The microphotograph **(i)** on the top illustrates a typical BDA-labeled terminal field in STN, as well as BDA-labeled passing axons in the adjacent internal capsule (IC). The microphotograph **(ii)** below is a high magnification of a portion of the BDA-labeled terminal field in STN showing a few axonal boutons (white arrows). Cd, caudate nucleus; GP, globus pallidus; OT, optic tract; Put, putamen; RT, reticular nucleus of the thalamus; SN, substantia nigra; Thal, thalamus.

The STN boundary was delineated using a series of 50 μm Nissl stained, frontal sections (at a total magnification of 12.5×), whereas the STN was scanned on a second (adjacent) series of sections processed for BDA, to chart and count the number of *terminal* or *en passant* axonal boutons (at a total magnification of 200×). The Nissl stained and the BDA sections were then superimposed, showing the spatial distribution of the axonal boutons in the STN (Figure 2). The size of the STN boutons was very small (below 1 μm in diameter) therefore forming a homogeneous population across all the animals. This is in contrast to the boutons observed in the different corticofugal projections, that exhibited various diameters (Fregosi and Rouiller, 2017; Fregosi et al., 2017, 2018, 2019). To account for various inter-section distances across animals and the analyzed number of sections of the BDA series, the raw numbers of boutons in each monkey were corrected by multiplying it by a correction factor, *f*:

$$f=\frac{d}{R*s}$$

where *d* is the inter-section distance in μm; *R* i*s* the reference (250 μm for all the animals) and *s* is the sampling interval (=0.5 if only one out of two sections were analyzed; or = 1 if all the sections were analyzed; Table 1). Finally, to compensate for variations in the size and location of the BDA injection sites, as well as differences in tracer uptake, the corrected numbers of boutons were divided by the number of corticospinal axons labeled just above the pyramidal decussation and multiplied by 1,000 to keep a realistic normalized number of boutons (Table 1). The normalization procedure has been described in detail earlier (Fregosi and Rouiller, 2017; Fregosi et al., 2017, 2018, 2019). Representation of the BDA injection sites in the 10 monkeys was published previously (Fregosi et al., 2017, 2018).

Due to the low number of animals we performed a bootstrapping analysis (MATLAB R2017b), where the initial population of *n* analyzed histological sections, covering the rostrocaudal extent of the STN, was resampled with replacement to obtain *k* = 100,000 fictive populations of *n* sections independently for each animal. For each bootstrapped population, we summed up the number of BDA-labeled boutons along the rostrocaudal axis to finally obtain the total number of BDA-labeled boutons of the 100,000 bootstrapped populations. As standard in bootstrapping, we calculated the *p*-value by estimating the residuals of the combined distribution testing for the null hypothesis that the gaussian distribution of the difference had a mean value of 0:

$$p=\int_{-\infty }^{0}N\left(\mu_{1}-\mu_{2},\frac{\sigma_{1}^{2}+\sigma_{2}^{2}}{2}\right)+\int_{0}^{+\infty }N\left(\mu_{2}-\mu_{1},\frac{\sigma_{1}^{2}+\sigma_{2}^{2}}{2}\right),$$

where *N* is the gaussian distribution function; *μ*_1_ and *μ*_2_ are the means of the two conditions with *μ*_1_ > *μ*_2_ and *σ*_1_ and *σ*_2_ are their respective standard deviations. Finally, the significance threshold of the *p*-value has been adjusted using the Bonferroni correction for multiple-group comparisons (*α* = 0.05 divided by the number of groups).

To assess the relationship between the number of BDA-labeled boutons with the interaction between motor impairment (PD symptoms based on manual dexterity in the modified-Brinkman board task post-ANCE transplantation (see above) and dopaminergic (tyrosine hydroxylase positive (TH+) neurons) loss in the substantia nigra pars compacta (SNpc), we performed a multivariable linear regression model test (MATLAB R2017b, function “fitlm”; Contestabile et al., 2018). To have a comparable (and reasonable amount of) variables between M1 and PM, the number of BDA-labeled boutons were normalized by dividing each variable by the maximum number of BDA-labeled boutons for M1 and PM, respectively. Note, that Mk-LL showed no PD symptoms in the clinical score (Borgognon et al., 2017) and in the reach and grasp drawer task (Borgognon et al., 2019). Therefore, we considered Mk-LL being at 100% of its motor performance although she had a hectic behavior in the modified-Brinkman board task (Table 1).

## Results

Following unilateral BDA injection in M1 or PM, labeled axons were visible in the homolateral internal capsule (mostly CST axons), together with a dark labeled axonal terminal field in STN (Figure 2Ai). At high magnification, the axonal terminal field contains identifiable axonal boutons (arrows, Figure 2Aii). The boutons were defined as a swelling of the axon branch, corresponding to at least a 2-fold increase in axon diameter (Fregosi and Rouiller, 2017; Fregosi et al., 2017, 2018, 2019). As shown in the Figure 2A for Mk-MY (MPTP intoxicated; BDA injected in PM), the BDA-labeled axonal boutons were located in specific subregions of the STN, whose position varied along the rostrocaudal axis. A comparable distribution of BDA-labeled axonal boutons is shown for two other representative monkeys, one MPTP intoxicated (Mk-MI; BDA injected in M1) and an intact animal (Mk-CH; BDA injected in PM; Figures 2B,C).

As shown in Figure 2A, the axonal boutons in STN were all tiny and smaller than axonal boutons observed in the ponto-medullary reticular formation (Fregosi et al., 2017, 2018) or in the superior colliculus (Fregosi and Rouiller, 2017; Fregosi et al., 2019). Indeed, the very small axonal boutons in STN were at the opposite extrema of the giant endings formed by another corticofugal projection originating in layer V, and terminating in the thalamus (e.g., Rouiller et al., 1998, 2003; Rouiller and Welker, 2000). As observed previously for the corticoreticular (Fregosi et al., 2017, 2018) and the corticotectal projections (Fregosi and Rouiller, 2017; Fregosi et al., 2019), in the STN the axonal boutons *en passant* were far more numerous than the boutons *terminaux*, although they could not always reliably distinguished, as previously reported (Fregosi et al., 2019).

**Figure 3:**
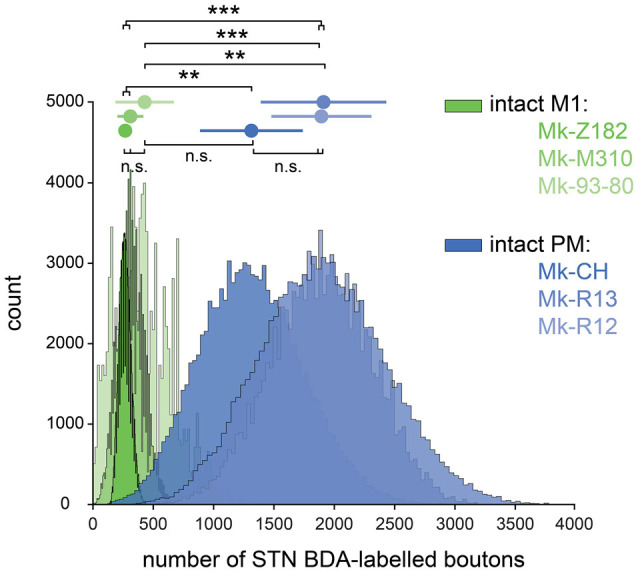
Bootstrapped distributions (see “Materials and Methods” section) with the mean (dot) ± standard deviation (bars) of the corrected and normalized numbers of corticosubthalamic boutons observed in STN in the six intact monkeys injected with BDA in primary motor cortex (green) and in premotor cortex (blue). Statistical analysis (see “Materials and Methods” section) comparing each animal with all the five others (six groups in total). Statistically significant differences (Bonferroni correction for multiple-group comparisons) are indicated as follows: **p < 0.0017, ***p < 1.6667e-04; “n.s.” refers to statistically non-significant (*p* > 0.0083).

The numbers of BDA-labeled axonal boutons over the sections covering the STN were then corrected, normalized, and finally bootstrapped, see “Materials and Methods” section. The final bootstrapped numbers of axonal boutons are shown in Figure 3 for monkeys injected with BDA in PM (blue) or in M1 (green). In each of the two subgroups of intact monkeys (PM or M1 injections), the three animals yielded comparable numbers of normalized axonal boutons in STN (statistically non-significant; *p* > 0.0083). However, the density of the CSTPs from PM was higher than those from M1 (*p* < 0.0083) in all two by two inter-animal comparisons, except Mk93-80 compared to Mk-CH (*p* = 0.0104).

**Figure 4:**
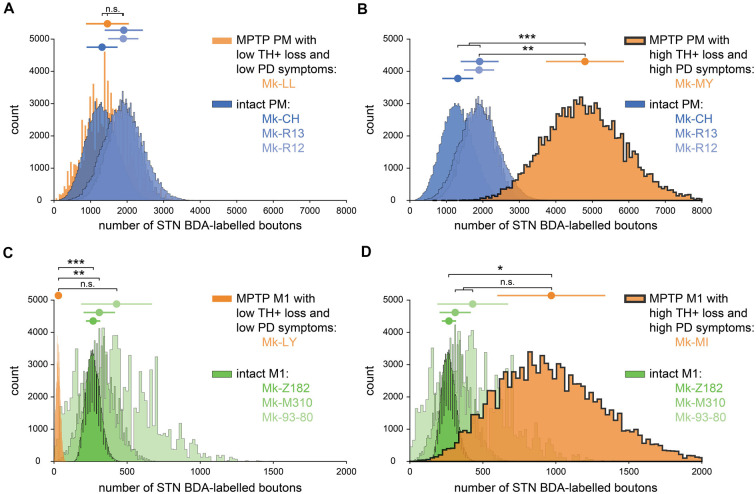
Bootstrapped distributions (see “Materials and Methods” section) with the mean (dot) ± standard deviation (bars) of the corrected and normalized numbers of corticosubthalamic boutons observed in STN in the 10 monkeys injected with BDA. **(A)** Comparison between the intact animals injected in PM (blue) with Mk-LL (MPTP animal injected in PM, orange). **(B)** Same as **(A)** but compared to Mk-MY. **(C)** Comparison between the intact animals injected in M1 (green) with Mk-LY (MPTP animal injected in M1, orange). **(D)** The same as **(C)** but compared with Mk-MI. **(A–D)** Statistical analysis (see “Materials and Methods” section) comparing each animal with all the three others (four groups in total). Statistically significant differences (Bonferroni correction for multiple-group comparisons) are indicated as follows: *p < 0.0125; **p < 0.0025, ***p < 0.00025; “n.s.” refers to statistically non-significant (*p* > 0.0125).

The inter-individual variability of the numbers of BDA-labeled axonal boutons in STN was larger in the PD monkeys than in intact monkeys, although again in the PD monkeys the projection from PM was stronger than from M1 (Table 1). More specifically, as a result of BDA injection in M1, one PD monkey (Mk-LY) exhibited a lower number of labeled axonal boutons in STN (Table 1, Figure 4C; *p* < 0.0125 except compared with Mk-93-80, where *p* = 0.0198). Mk-LY was characterized by a modest loss of TH+ neurons in the SNpc (−39%), and a complete spontaneous functional recovery (see Borgognon et al., 2017). The second PD monkey (Mk-MI; subjected to BDA injection in M1) exhibited about twice the number of labeled axonal boutons in STN, as compared to the three intact animals (Table 1), although the bootstrap analysis showed a significant difference only in animal Mk-Z182 (Figure 4D, *p* = 0.0075). Mk-MI was characterized by a significant loss of dopaminergic neurons in SNpc (−74%) and severe PD symptoms. In the two PD monkeys subjected to BDA injection in PM, one animal (Mk-LL, −67% of TH+ neuronal loss) had numbers of labeled axonal boutons in STN that were comparable to intact animals (Figure 4A, *p* > 0.0125), whereas the second monkey (Mk-MY, −72% of TH+ neuronal loss) showed a dramatically higher number of axonal boutons in STN (Figure 4B, *p* < 0.0125).

**Figure 5:**
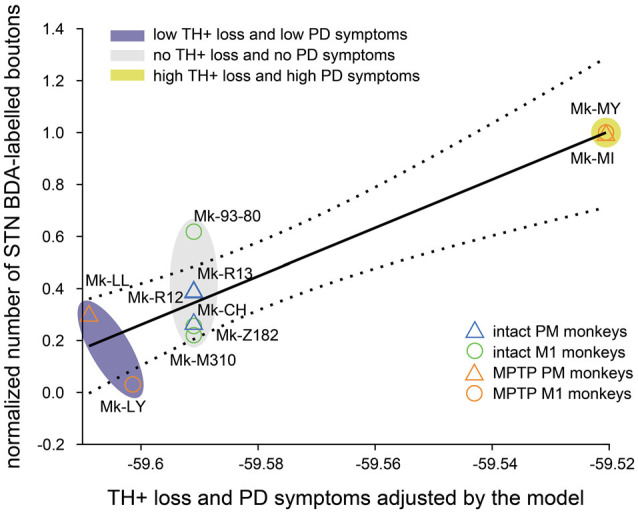
Multivariable linear regression model (see “Materials and Methods” section). Fit (solid black line) and the 95% confidence bounds (black dashed lines) of the constant (number of BDA-labeled boutons) vs. the other variables (PD symptoms and TH+ loss in SNpc) in the model. The multi variables are “partialed out” but not the constant term. Therefore, the X-abscissa represents the combined independent variables (PD symptoms and TH+ loss) adjusted by the model. The model has an explanatory power (positive slope: *y* = 9.298*x; *R*^2^ = 0.821; adjusted *R*^2^ = 0.731; *p*-value = 0.0117). Three clusters (ovals) appear: (1) the highest TH+ loss and motor impairment (yellow); (2) the lowest loss of TH+ neurons and motor impairment (purple) and (3) the intact animal cluster (gray) without dopaminergic loss nor PD symptoms.

Predictably, the summary of the multivariable linear regression model showed a significant interaction between TH+ loss in SNpc and motor impairment (Table 2, *p* = 0.015). To visualize the fit of the dependent variables (number of boutons) against the independent variables (TH+ loss and PD symptoms), all the variables were “partialed-out” except for the constant term. The model showed an explanatory power (positive slope: *y* = 9.298*x). If it did not, it would appear as a horizontal line (Figure 5). Moreover, the model showed a positive correlation between the number of BDA-labeled boutons with the loss of TH+ neurons in SNpc and the motor impairment (root mean squared error = 0.169; *R*^2^ = 0.821, adjusted *R*^2^ = 0.731, *p*-value = 0.0117). The four PD monkeys were clustered in two different spaces compared to the intact group. Indeed, the two PD animals (Mk-LY and Mk-LL) with less TH+ loss and the lowest motor impairment, were located at the bottom left of the graph (purple cluster), whereas the two PD animals (Mk-MY and Mk-MI) with more TH+ loss and greater motor impairment were on the top right of the graph (yellow cluster).

**Table 2:** Estimated coefficients of the linear regression model.

	Estimate	SE	tStat	p-value
Intercept	554.45	163.72	3.387	0.015
TH+ loss in SNpc	−7.47	2.21	−3.377	0.015
PD symptoms	−5.54	1.63	−3.385	0.015
TH+ loss : PD symptoms	0.07	0.02	3.378	0.015

## Discussion

Although the topography of the CSTPs from M1 and PM in macaques has been established previously (Nambu et al., 1997), the originality of the present study is to provide new evidence that the projection from PM to STN is denser than that from M1 to STN, after normalization, based on the number of labeled CST axons. Recently, Coudé and colleagues have shown that the innervation of the STN arises from the collaterals of long-range corticofugal axons *en route* to lower brainstem regions. In our analysis, we used the numbers of CST axons to normalize the data, which could bias the results if CST neurons emit collaterals to the STN. However, the bias would be in favor of M1 projections (more boutons from M1 than PM) because ~50% of CST neurons originate from M1 as opposed to only ~10–20% from PM (Dum and Strick, 1991). A similar difference between PM and M1 was found for the corticoreticular projection (Fregosi et al., 2017), as well as the corticotectal projection (Fregosi and Rouiller, 2017). In other words, in the sense of a tentative generalization, it seems that PM is in a position to exert a stronger influence on subcortical relays (STN, ponto-medullary reticular formation, superior colliculus) than M1. As far as the topography of the axonal terminal field in STN is concerned, the present data are largely consistent with the study of Nambu et al. (1997).

The quantitative data of the present study depend strongly on the normalization procedure based on the number of BDA-labeled CST axons in the same monkeys. The pros and cons of such normalization were discussed in detail earlier (Fregosi and Rouiller, 2017; Fregosi et al., 2017, 2018, 2019). The rationale for such normalization is to compensate for variability in tracer uptake and injection site sizes. In particular, to take into account variations in the laminar spread of BDA in the injected area, with the consideration that layer V is the most relevant to the investigation of corticofugal projections to the basal ganglia, the ponto-medullary reticular formation, or the superior colliculus (Gerfen et al., 2018). The number of CST axons can easily be counted above the pyramidal decussation and as the CST axons also originate from layer V, it may be an adequate normalizing factor to compensate for interindividual differences in terms of BDA spread in layer V.

The present data suggest that the CSTP is increased in PD monkeys subjected to the ANCE treatment, although this observation is restricted to two out of four animals. The limitation here is of course the interindividual variability among the PD monkeys, as well as the low number of cases, which is, however, often the case in non-human primate studies for ethical reasons. Obviously, the present pilot study needs to be extended with more monkeys (intact and PD), especially PD monkeys without ANCE treatment (see below). The increase of the CSTP in the two PD monkeys (Mk-MY and Mk-MI) is in apparent contradiction with previous reports of a reduction of the cortical innervation of the STN in PD monkeys (Mathai et al., 2015) and in PD mice (Chu et al., 2017). The discrepancy may result from the fact that the present PD monkeys were treated with ANCE, whereas there were no treatments in the other two studies (Mathai et al., 2015; Chu et al., 2017). However, the two PD monkeys with less dopaminergic neuronal loss and low motor impairment (Mk-LY and Mk-LL, purple cluster) are more in concordance with the two previous studies (Mathai et al., 2015; Chu et al., 2017). Indeed, Mk-LY showed a dramatic decrease in the CSTP from M1, whereas Mk-LL exhibited comparable CSTP from PM. Overall, the linear model may suggest that the re-organization of the CSTP depends on both the PD symptoms and the dopaminergic loss in SNpc. Again, future experiments are needed in PD monkeys subjected to BDA injections in PM or M1, but in absence of ANCE treatment, with the hypothesis that the CSTP may decrease from M1 and remain stable from PM, as compared to intact monkeys. Furthermore, the readout for axon terminals in STN was not the same across studies (Mathai et al., 2015; Chu et al., 2017 vs. present study; see “Introduction” section). In the same four PD monkeys treated with ANCE, the corticoreticular projection was reduced as compared to intact monkeys (Fregosi et al., 2018). The present study suggests changes in the CSTP in cases with PD-like symptoms in ANCE treated monkeys, although, with individual variability, it is consistent with the notion that the STN is a major target for the reduction of PD symptoms, as has been demonstrated in MPTP monkeys (e.g., Benazzouz et al., 1993; Guridi et al., 1994, 1996).

The increase of the CSTPs observed in two out of four PD monkeys, when compared to the percentage of loss of dopaminergic neurons (TH+ neurons) in the SNpc, may be indicative of a threshold effect. Indeed, it is known that stable PD symptoms remain present after MPTP intoxication only when a threshold is reached for the loss of dopaminergic neurons in SNpc (see e.g., Soderstrom et al., 2006; Borgognon et al., 2017). In the present report, the increase of CSTPs was observed in the two PD monkeys with a greater than 70% loss of dopaminergic neurons (Mk-MY and Mk-MI), whereas in the other two PD monkeys the dopaminergic neurons’ loss was lower than 70%. A tentative (and speculative) threshold at 70% for an effect on the CSTP would be consistent with the behavioral observation that the monkeys Mk-MY and Mk-MI exhibited stronger and more stable motor deficits after MPTP lesion, and before ANCE treatment, than the other two PD monkeys (Borgognon et al., 2017, 2019).

## Data Availability Statement

On request, access can be provided to the histological sections (contact the corresponding author).

## Ethics Statement

The animal study was reviewed and approved by Service de la sécurité alimentaire et des affaires vétérinaires SAAV Amt für Lebensmittelsicherheit und Veterinärwesen LSVW.

## Author Contributions

EMR designed the tracing experiments. EMR analyzed the histological sections. SBo performed the multivariable linear model and the bootstrapping analysis. SBa, JC, EMR and SBo designed and performed the MPTP experiments. JB and J-FB designed the ANCE treatment. EMR and SBo drafted the manuscript. All authors contributed to the article and approved the submitted version.

## Conflict of Interest

The authors declare that the research was conducted in the absence of any commercial or financial relationships that could be construed as a potential conflict of interest.

## Abbreviations

CSTP: corticosubthalamic projection
BDA: biotinylated dextran amine
CST: corticospinal
MPTP: 1-methyl-4-phenyl-1,2,3,6-tetrahydropyridine
PD: Parkinson-like symptoms
M1: Primary motor cortex
PM: Premotor cortex
SNpc: substantia nigra pars compacta
STN: subthalamic nucleus
ANCE: autologous neural cell ecosystem.
